# CHATGPT ANALYSIS OF AGEISM IN THE 2024 PRESIDENTIAL ELECTION: RESEARCH, POLITICS, AND POLICY IMPLICATIONS

**DOI:** 10.1093/geroni/igae098.3828

**Published:** 2024-12-31

**Authors:** Faith Hopp

**Affiliations:** Wayne State University, Detroit, Michigan, United States

## Abstract

Gerontologist Robert Butler introduced the concept of Ageism, described as “the great sleeper in American life” in a 1968 interview with journalist Carl Bernstein. Ageism refers to discrimination based on age and has been particularly evident during the 2024 election season. To further examine this relevant phenomenon, we used ChatGPT to analyze instances of ageism related to President Biden’s campaign. We instructed ChatGPT to collect a sample of articles from mainstream media mentioning President Biden (N=23), asking the program to score the frequency and type of ageist language during the month before and after the June 27 2024 Biden-Trump debate. Ageist themes included statements about Biden being a bad leader, not trustworthy, not mentally and/or physically fit, and not competent and/or relevant as a candidate. The number of ageist statements about Biden increased in the month following the debate compared with the pre-debate month. We found fewer ageist-related statements about Biden in the African American press both before and following the debate, but access to these articles was limited due to paywall restrictions. These findings suggest that ageism continues to be relevant and will continue to drive age-related discourse, impacting policy, practice, and older adult life experiences. Future researchers should consider the role of internet paywalls in limiting public access to news sources and the breadth of content available for ChatGPT analysis. We recommend that researchers obtain full-text articles using newspaper databases rather than relying on ChatGPT internet scans to ensure comprehensiveness and analytical depth.

